# An evaluation of adverse events following an immunization campaign with the live, attenuated SA14-14-2 Japanese encephalitis vaccine in Cambodia

**DOI:** 10.1371/journal.pone.0269480

**Published:** 2022-06-09

**Authors:** Susan L. Hills, Sann Chan Soeung, Svay Sarath, Chheng Morn, Cheam Dara, Marc Fischer, Michael C. Thigpen

**Affiliations:** 1 Arboviral Diseases Branch, Centers for Disease Control and Prevention, Fort Collins, Colorado, United States of America; 2 National Immunization Program, Ministry of Health, Phnom Penh, Cambodia; 3 Consultant, Phnom Penh, Cambodia; 4 PATH, Phnom Penh, Cambodia; PLOS, UNITED KINGDOM

## Abstract

**Introduction:**

Japanese encephalitis (JE) virus is the most common cause of vaccine-preventable encephalitis in Asia. The SA14-14-2 JE vaccine manufactured by Chengdu Institute of Biological Products has been shown to be safe and effective in clinical trials and childhood routine immunization programs. However, there are few published reports describing results of surveillance for adverse events following immunization (AEFI) when the vaccine is used in mass campaigns. We describe the results of AEFI surveillance following a 2013 vaccination campaign among almost 310,000 children aged 9 months–12 years in Battambang Province, Cambodia.

**Methods:**

Routine AEFI surveillance was strengthened by staff training and supplemented by active hospital surveillance. An AEFI was defined as any sign, symptom, or disease temporally associated (i.e., within 4 weeks) with receipt of the vaccine, irrespective of whether it was considered related to immunization. Data were collected on standardized forms and causality assessments were conducted for serious AEFI.

**Results:**

Passive and active surveillance detected 28 AEFI for an overall incidence of 9.0 AEFI per 100,000 doses administered. The most frequent events were vasovagal episodes (n = 7, 25%) and rash (n = 6, 21%), and most other events were common childhood conditions such as fever and vomiting. Three AEFI were classified as serious, including one hypersensitivity reaction and two meningoencephalitis cases. Of these, the hypersensitivity event was the only serious AEFI classified as being consistent with a causal association to immunization.

**Conclusions:**

Most reported adverse events were conditions that commonly occur after other childhood vaccinations or independently of vaccination, and in the context of careful monitoring for serious AEFI only one serious event consistent with a causal association with immunization was identified. These results support the good safety profile of the SA14-14-2 JE vaccine, and provide reassuring data as the vaccine’s use expands.

## Introduction

Japanese encephalitis (JE) virus, a mosquito-borne flavivirus, is the most common cause of vaccine-preventable encephalitis in Asia [1, 2]. Globally, an average of 67,900 clinical cases of JE and up to 20,400 deaths are estimated to occur annually [2]. Approximately 30%–50% of survivors have long-term neuropsychological sequelae.

The World Health Organization (WHO) recommends that JE vaccination should be integrated into national immunization programs in all areas where JE is recognized as a public health priority [3]. The live attenuated SA14-14-2 JE vaccine manufactured by Chengdu Institute of Biological Products (trade name CD.JEVAX) has been available in China since 1988. More recently, WHO prequalification, improved international availability, and Gavi funding for JE vaccination campaigns have led to a substantial increase in use in other Asian countries [1, 4, 5]. The vaccine has been shown to be safe and effective in clinical trials and when used in childhood routine immunization programs [3, 6–10]. However, there are few published reports describing surveillance for adverse events following immunization (AEFI) when SA14-14-2 JE vaccine has been used in large immunization campaigns with vaccine administered to children in a broad age range [11, 12]. The WHO’s Global Advisory Committee on Vaccine Safety has recommended that campaigns should be accompanied by strong AEFI monitoring and investigation activities [11]. Availability of AEFI data are important to maintain public confidence in vaccine safety and immunization programs, which in turn is critical for ensuring high vaccination coverage and effective disease control [13].

A JE immunization campaign for children aged 9 months through 12 years was conducted in Battambang Province, Cambodia in 2013. A JE vaccination program had been implemented in three other Cambodian provinces in 2009, with vaccine delivered through the routine immunization program to children aged 10–24 months, but no mass JE vaccination campaign previously had been conducted. During the campaign, 309,507 children were vaccinated with SA14-14-2 JE vaccine. Routine passive AEFI surveillance was strengthened by staff training and supplemented by active hospital surveillance to ensure sensitivity of reporting. We describe the results of this surveillance to provide data on vaccine safety from a large campaign involving children from infants to adolescents.

## Methods

### JE immunization campaign

The Cambodia Ministry of Health conducted a JE immunization campaign in Battambang Province from February 18 through March 14, 2013 using Expanded Program on Immunization staff who had conducted previous mass immunization campaigns for other vaccines. Children were vaccinated with one 0.5mL subcutaneous dose of SA14-14-2 JE vaccine. The vaccine was supplied in five-dose vials and no other vaccines or medications were administered as part of the campaign.

### Case definitions and classifications

An AEFI was defined as any sign, symptom, or disease temporally associated (i.e., within 4 weeks) with receipt of the vaccine, irrespective of whether it was considered related to immunization. An adverse event was classified as a hypersensitivity reaction if any of the major or minor dermatologic/mucosal or respiratory criteria of the Brighton Collaboration case definition for anaphylaxis were present [14, 15]. Meningoencephalits was defined according to the standard definition used for routine surveillance purposes in Cambodia, based on a modified version of the WHO case definition for acute encephalitis syndrome [16]. A clinical meningoencephalitis case was defined as a person with the sudden onset of fever with one or more of the following: 1) change in mental status, 2) new onset of seizures, movement disorders, or flaccid paralysis, or 3) neck stiffness or other meningeal signs. A serious AEFI was defined as any event requiring inpatient hospitalization for ≥1 night.

### Passive surveillance

Vaccinators and supervisors attended training during the month prior to the campaign’s commencement. The training reinforced the processes of the Cambodia Ministry of Health’s routine AEFI reporting system, including the approach to investigation and reporting of events, and operational field guidelines were distributed. During the campaign, vaccinators and healthcare workers recorded AEFI that were observed by them directly, came to them via phone or word of mouth from Village Health Support Group staff, teachers, parents or others, or resulted in a child visiting a healthcare center. Cambodia’s standardized AEFI reporting form was used to collect information that included patient demographics, date, time and place of vaccination, a clinical description of the event, date and time of onset, and the vaccine lot number and expiry date. AEFI forms were collected daily by a district or provincial-level supervisor and forwarded to the provincial immunization program manager. For any serious AEFI, provincial-level staff conducted an additional investigation.

### Active surveillance

During the campaign, active surveillance for serious AEFI was conducted at three of four hospitals in Battambang province. International and national supervisors checked for AEFI at each hospital every 1–7 days. Pediatric ward registers were reviewed and staff were questioned about whether any child who reported JE vaccination had been admitted. During the 4 weeks following the campaign, two site visits were conducted to all four hospitals. Additionally, records were reviewed at a hospital in an adjacent province where sick children were sometimes referred.

### Laboratory testing for meningoencephalitis cases

Routine testing for meningoencephalitis cases conducted in Cambodia included bacterial culture, a Gram stain, and a Ziehl-Neelsen stain on cerebrospinal fluid (CSF), and a complete blood count. Additional laboratory testing was conducted at the US Centers for Disease Control and Prevention (CDC) to investigate possible etiologies. Testing was limited by the type and amount of sample available but tests used included the CDC JE immunoglobulin M (IgM) enzyme-linked immunosorbent assay (ELISA), the CDC dengue IgM ELISA, the Panbio JE-dengue IgM combo ELISA (manufactured by Inverness Medical Innovations, Australia), and real-time reverse transcription–polymerase chain reaction (rRT-PCR) testing for JE virus, *Neisseria meningitidis*, *Haemophilus influenzae* type B, and *Streptococcus pneumoniae*.

### Causality assessment for serious AEFI

Two team members (SH and MT) independently assessed serious AEFI reports; the national AEFI causality committee was unable to convene at the time of the campaign. Events were classified according to WHO guidelines as 1) consistent with a causal association with immunization, 2) indeterminate (consistent temporal relationship but insufficient evidence for causality or conflicting trends of consistency and inconsistency with causality), or 3) inconsistent with a causal association to immunization [17].

### Data analysis

We reviewed AEFI reports for patient age and sex, interval between vaccination and symptom onset, vaccine information, and for the description of the event. Categorical variables were described as counts and proportions, and continuous variables were described by median and range. We calculated incidence of AEFI per 100,000 doses administered.

### Ethical considerations

Consent for immunization was not required as this was a routine immunization campaign and not a research activity. All activities were approved by the Cambodian Ministry of Health, and the US Centers for Disease Control determined that the activity did not meet the definition of research under 45 CFR 46.102 and therefore review by an Institutional Review Board was not required. Additional information regarding the ethical, cultural, and scientific considerations specific to inclusivity in global research is included in the Supporting Information (S1 Checklist).

## Results

### AEFI reported through passive and active surveillance

Twenty-six adverse events were reported through passive AEFI surveillance and two additional events were detected through active hospital surveillance, for an overall incidence of 9.0 AEFI per 100,000 doses administered. Fourteen (50%) of the 28 events were in females, 11 (39%) in males, and three reports did not include the child’s sex. The median age was 5 years (range 1‒12 years). Adverse events occurred a median of 7 hours after vaccination (range 0 hours‒10 days).

Among the 28 AEFI, the most frequent events were vasovagal episodes (n = 7, 25%) and rash (n = 6, 21%) (**Table 1**). Two (7%) events were classified as hypersensitivity reactions and two (7%) as meningoencephalitis. Three (11%) AEFI were classified as serious (1 per 100,000 doses), including one of the hypersensitivity reactions and the two meningoencephalitis cases.

**Table 1:** Non-serious and serious adverse events following administration of SA14-14-2 JE vaccine to 309,507 children aged 9 months–12 years during an immunization campaign in Cambodia, 2013.

AEFI	Non-serious (N = 25)		Serious (N = 3)		Total (N = 28)	
	No.	(%)	No.	(%)	No.	(%)
Vasovagal episode	7	(28)	0	(0)	7	(25)
Rash	6	(24)	0	(0)	6	(21)
Fever^a^	3	(12)	0	(0)	3	(11)
Meningoencephalitis	0	(0)	2	(67)	2	(7)
Hypersensitivity reaction^b^	1	(4)	1	(33)	2	(7)
Vomiting	2	(8)	0	(0)	2	(7)
Diarrhea	1	(4)	0	(0)	1	(4)
Crying/cyanosis	1	(4)	0	(0)	1	(4)
Pharyngitis	1	(4)	0	(0)	1	(4)
Acute respiratory infection	1	(4)	0	(0)	1	(4)
Submandibular gland infection	1	(4)	0	(0)	1	(4)
Epistaxis	1	(4)	0	(0)	1	(4)

^a^Fever without additional symptoms or signs

^b^One case of generalized urticaria and one of angioedema

### Hypersensitivity reactions

**Fig 1:**
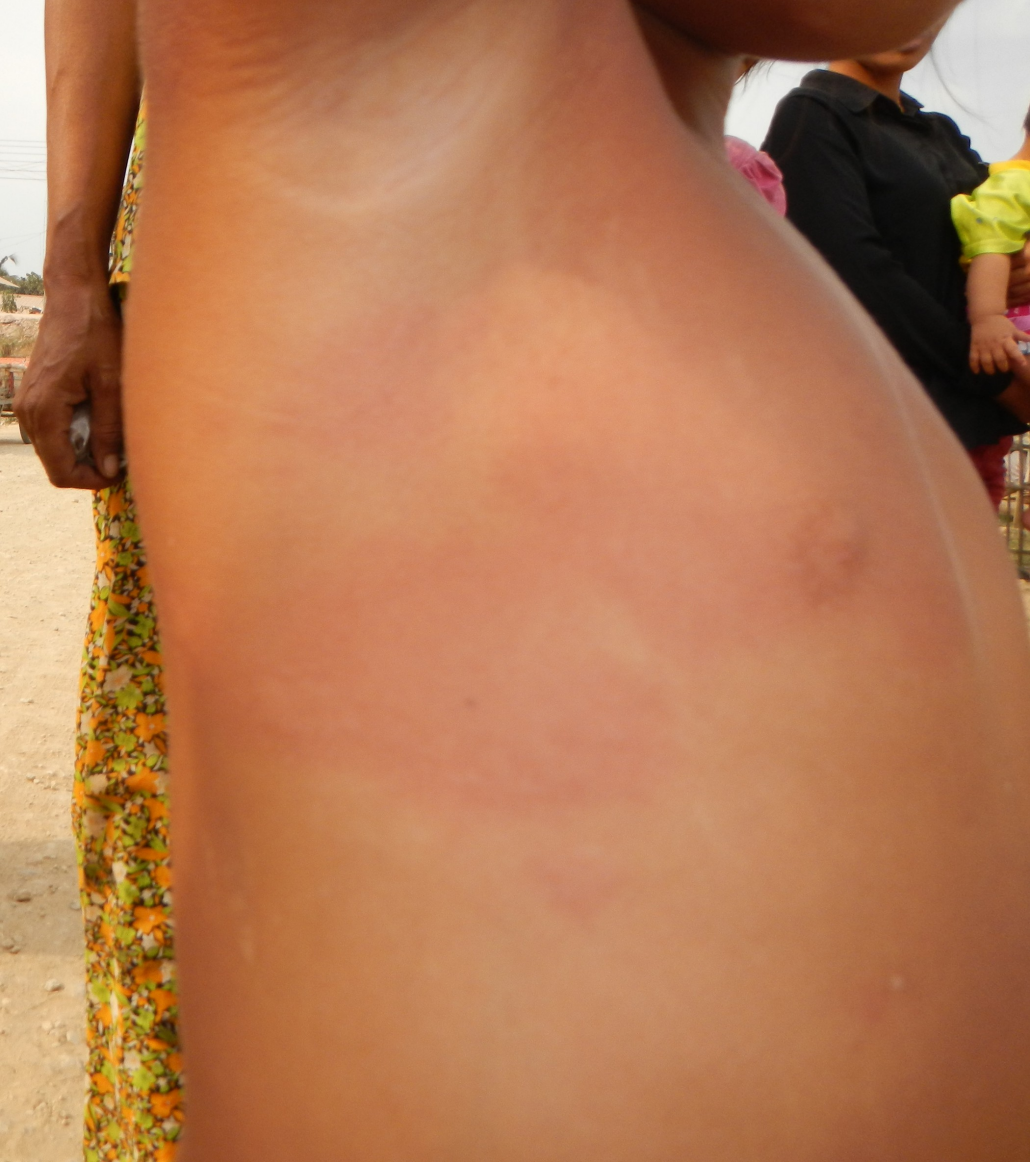
Generalized urticaria in a 5-year-old child which appeared at 25 hours following vaccination.

One non-serious hypersensitivity reaction occurred in a 5-year-old female who developed generalized urticaria and fever at 25 hours after vaccination; she was treated with topical medications and did not require hospitalization (**Fig 1**). The serious hypersensitivity reaction occurred in an 11-year-old female. She developed angioedema with fever and vomiting 45 hours following vaccination. She was hospitalized for 4 days, treated with chlorpheniramine and intravenous hydrocortisone, and fully recovered. Based on the temporal relationship and biological plausibility, this serious AEFI was classified as “consistent with a causal association with immunization”.

An additional six AEFI reports could have been hypersensitivity reactions as they described rashes following immunization, but the reports provided insufficient detail for this classification. The median age of the six children was 5 years (range: 1–10 years) and the median interval between vaccination and rash onset was 8 hours (range: 20 minutes–4 days). Two reports described the rash occurring with fever, and one described the rash as being itchy. Two children received medications typically used to treat allergic reactions.

### Meningoencephalitis cases

Two reports among vaccinated children met the clinical case definition for meningoencephalitis; an additional case was reported but was determined to be a child aged 2 months and too young for vaccination. A 3-year-old female developed headache, fever, neck stiffness, seizures, and confusion 9 days after vaccination and was admitted to hospital 2 days later. CSF collected on admission showed pleocytosis (55 white blood cells (WBC)/μL with 67% polymorphonuclear neutrophils and 30% lymphocytes), 3 red blood cells/μL, elevated protein (86 mg/dL [normal: 5–40 mg/dL]), and low glucose concentration (19 mg/dL [normal: 40–80 mg/dL]). Her peripheral WBC count showed leukocytosis (45,000 WBC/μL with 80% neutrophils). The child was treated with broad spectrum antibiotics, but died the following day. CSF was negative for JE virus-specific IgM antibodies by the CDC ELISA and the Panbio JE-dengue IgM combo ELISA and JE virus ribonucleic acid also was not detected. Serum was positive for JE IgM antibodies by Panbio JE-dengue IgM combo ELISA but negative by CDC ELISA. CSF and serum were negative for dengue virus-specific IgM antibodies. Bacterial culture and gram stain of CSF were negative, and no acid-fast bacilli were noted. No other testing was possible due to depletion of the samples. Due to the absence of JE IgM antibodies or JE ribonucleic acid in the CSF, this event was classified as “inconsistent causal association to immunization (coincidental)”.

The second case was in a 4-year-old male who developed fever, neck stiffness, seizures, hematemesis, abdominal pain, and a non-pruritic rash on his face and arms at 10 days after receiving JE vaccine. On admission to hospital 2 days later, he was febrile (38.8°C) and unconscious. Blood was collected on admission and his peripheral WBC count was 39,100 WBC/μL with 93% neutrophils. No lumbar puncture was performed. He was commenced on ceftriaxone, paracetamol, and diazepam but his condition deteriorated and he died 4 hours later. Serum was positive for JE IgM antibodies by Panbio JE-dengue IgM combo ELISA but negative by CDC ELISA. Serum was negative for dengue IgM antibodies. Serum was also negative by rRT-PCR for *Neisseria meningitidis*, *Haemophilus influenzae* type B, and *Streptococcus pneumoniae*. Because limited testing could be done to identify possible etiologies, this event was classified as “indeterminate”.

## Discussion

Results from AEFI surveillance conducted during this large JE immunization campaign among >300,000 children support the safety of SA14-14-2 JE vaccine. Passive surveillance for all AEFI and active surveillance for serious AEFIs detected 28 AEFI for an overall incidence of 9.0 AEFI per 100,000 doses administered to children aged 9 months–12 years. The AEFI that occurred were mostly conditions that commonly occur after other childhood vaccinations or independently of vaccination, including rash, fever, and vasovagal events, and most events were considered non-severe. Among three serious AEFI occurring in temporal association with vaccine administration, only one, a hypersensitivity event, was classified as causally related to vaccination. With mostly common conditions reported and only one non-fatal vaccine-related serious AEFI in the context of careful monitoring for such events, these data are reassuring regarding the vaccine’s safety.

Safety data from clinical trials showed SA14-14-2 JE vaccine to be well-tolerated and established the vaccine’s good safety profile [3, 6, 7, 10, 18]. Available post-marketing surveillance data primarily have been collected through routine passive AEFI surveillance systems and during the vaccine’s use in routine immunization programs [19–21]. It is difficult to compare AEFI rates from post-marketing surveillance conducted in diverse settings with different methodologies, and when SA14-14-2 JE vaccine is used in routine immunization programs when it is often co-administered with measles or other vaccines. However, no important safety concerns have been identified [3, 11, 18]. A strength of the data reported here are that they were collected through passive and active surveillance, during a short period following administration of almost 310,000 vaccine doses in one province, and during a campaign in which close attention was given to AEFI surveillance, likely increasing the sensitivity of event detection.

Hospital surveillance detected two case of meningoencephalitis that occurred in children 9–10 days following immunization. Extensive laboratory testing confirmed one was not related to JE vaccination, with CSF findings of a low glucose concentration suggesting a bacterial, fungal or parasitic cause of infection. For the second patient, lack of CSF limited possible laboratory testing, but clinical findings including hematemesis and a rash on the face and arms, and laboratory findings of a peripheral neutrophilic leukocytosis, suggested the event was probably unrelated to JE vaccination. It is not surprising that these two cases, one unrelated and one likely unrelated to vaccination, were identified among the population of vaccinated children, given the campaign coverage rate was estimated to be 91% (M. Thigpen, pers. comm). It is important to monitor for neurological events as part of comprehensive AEFI surveillance, but when large numbers of children are vaccinated during campaigns, coincidental meningoencephalitis cases are likely to occur. Thorough investigation is important to avoid incorrectly attributing causality for temporally associated events to vaccination, and a comprehensive investigation may provide an alternate etiology. For example, a case report from India described a child who presented with neurological symptoms 3 days following receipt of SA14-14-2 JE vaccine, and subsequent testing indicated chikungunya virus infection [22]. Samples collected for laboratory testing should include CSF, whenever possible. Detection of JE IgM antibodies in serum following vaccination with SA14-14-2 JE vaccine is not unexpected, with up to 44% of children reported to have a JE IgM positive or equivocal result within 1 month of vaccination, so antibody detection in serum does not help to clarify whether an AEFI might be vaccine-related [23]. For both children with meningoencephalitis detected during surveillance activities following the campaign, discrepant JE IgM results were reported when serum was tested by the Panbio and CDC ELISAs, which is not uncommon with assays with different sensitivities and specificities [23, 24]. Regardless, the result would not have contributed to understanding each event’s etiology. For fatal cases, an autopsy should be performed if feasible.

This report is subject to several limitations. Underreporting of AEFI likely occurred, particularly non-serious events, and children with AEFI might have presented to hospitals in other provinces; we reviewed records at the primary pediatric referral hospital in an adjacent province, but did not have access to records at hospitals in other provinces. In addition, parents might not have sought care for their children at a health facility if an AEFI occurred; studies have shown that Cambodian villagers might not seek care within the public healthcare system, even for severe disease [25]. Finally, some reports were incomplete, such as six reports of rash that did not include sufficient clinical information to enable the event’s inclusion or exclusion as a hypersensitivity reaction.

These data, collected during a mass vaccination campaign among a wide age range of children, provide additional information to support the vaccine’s good safety profile and are reassuring in the context of expanded use of SA14-14-2 JE vaccine. However, as recommended by WHO, careful monitoring for AEFI and complete investigation of serious events should always accompany immunization programs [11].

## Supporting information

S1 Checklist Inclusivity in global research questionnaire. (PDF)

S1 Dataset Information on children with an adverse event following immunization. (PDF)
