# Supplementary material for: An evaluation of adverse events following an immunization campaign with the live, attenuated SA14-14-2 Japanese encephalitis vaccine in Cambodia
Source: PLoS One. 2022 Jun 9;17(6):e0269480. doi: 10.1371/journal.pone.0269480 (PMC9182652; doi:10.1371/journal.pone.0269480)
Supplement: S1 Dataset — (PDF) [file pone.0269480.s002.pdf]

S2. Information on children with an adverse event following immunization				
**Number**	**Age**	**Sex**	**Interval (hours)**	**Event**
1			0	Vasovagal
2				Crying/cyanosis
3	11	M	<1	Vasovagal
4	4	M	1	Fever
5				Vasovagal
6	6	M	11	Submandibular glandular swelling
7	9	M	<1	Vasovagal
8	6	F	<1	Vomitting
9	4	M	1	Fever
10	7	F	<1	Vasovagal
11	8	M	<1	Vomitting
12	5	F	25	Pharyngitis
13	11	M	0	Vasovagal
14	5	F	25	Hypersensitivity
15	1	F	29	Fever
16	11	F	10	Epistaxis
17	11	F	45	Hypersensitivity
18	5	F	<1	Rash
19	2	M	6	Rash
20	2	F	124	Diarrhea
21	10	M	96	Rash
22	1.8	F	94	Rash
23	5	F		Rash
24	7	F	8	Rash
25	12	F	<1	Vasovagal
26	3	M	Same day	Acute respiratory infection
27	3	F	216	Meningoencephalitis
28	4	M	240	Meningoencephalitis
